# A village health worker intervention to reduce cardiovascular disease risk in remote areas of armed conflict in Myanmar–results from a feasibility study in three villages

**DOI:** 10.1186/s13031-026-00785-2

**Published:** 2026-03-15

**Authors:** Anu Ramachandran, Su Mon Thwe, Cho Zin Win, Nay Lynn Htet, Saw Kyaw Myint, Nan Ei Mon Myint, Nicholus Tint Zaw, Ravi Goyal, Zin Mar Win, Zay Yar Phyo Aung, Tom Traill, Adam Kimball Richards, Parveen Parmar

**Affiliations:** 1 Center for Innovation to Implementation, VA Palo Alto, Palo Alto, CA USA; 2 Department of Health Policy, Stanford University, Stanford, CA USA; 3 Karen Department of Health and Welfare, Klo Yaw Lay, Karen State Myanmar; 4 Organization name withheld for security reasons, Chiang Mai, Thailand; 5 Organization name withheld for security reasons, Berkeley, CA USA; 6 University of California, San Diego, CA USA; 7 Center for Humanitarian Health, Department of International Health, Bloomberg School of Public Health, Johns Hopkins University, Baltimore, MD United States; 8 Mahidol University, Bangkok, Thailand; 9 Department of Emergency Medicine, Harbor–UCLA Medical Center, Torrance, United States; 10 Department of Emergency Medicine, University of California, Los Angeles David Geffen School of Medicine, California, United States

**Keywords:** Community health worker, Armed conflict, Feasibility study, Myanmar, Cardiovascular disease

## Abstract

**Background:**

Cardiovascular disease (CVD) is a leading cause of death in low-income countries and those affected by armed conflict, including Myanmar. Community health worker interventions can effectively address CVD risk factors in low-income countries but have not been tested among displaced populations in active conflict zones.

**Objectives:**

We conducted a feasibility study of a village health worker (VHW) care model to identify individuals at high CVD risk and deliver care in conflict-affected regions of Karen State, Myanmar. This study was conducted by an international non-governmental organization collaborating with a regional local health organization.

**Methods:**

Following a village census, trained VHWs and medics screened individuals age ≥ 40 for CVD risk factors in three villages. Eligible individuals had HTN, diabetes, calculated CVD risk > 10%, or history of heart attack or stroke, confirmed during a second visit 1–2 weeks later. VHWs visited households every 3–6 weeks for 2 months to monitor blood pressure, glucose, medication adherence/side effects, and deliver medic-prescribed medications. Feasibility evaluation centered on reach, adoption, and acceptability. Outcomes included CVD risk factor prevalence, recruitment and retention, medication initiation/adherence, changes in hypertension control, and adverse outcomes. VHW and medic focus group discussions and study participant interviews were conducted.

**Results:**

CVD teams screened 294 individuals, conducted confirmatory visits with 132, enrolled all 97 eligible participants, and completed two home visits with 94 patients. Several prescription errors were made, halting medication initiation; root cause analysis identified opportunities to improve pre-testing of electronic tools and strengthen clinician CVD training. The proportion of eligible participants receiving antihypertensive or statin medications increased from 23% to 56%. Among those with HTN, the proportion achieving blood pressure control < 140/90 mmHg increased from 22.9% to 65.7%. Qualitative assessment revealed support for the care model and opportunities to improve training and streamline clinical protocols.

**Conclusions:**

Our study suggests a VHW care model for CVD in remote villages in Myanmar experiencing armed conflict is feasible and can increase medication access. Opportunities exist to simplify CVD treatment guidelines and augment training and support of local providers. Findings informed a cluster randomized controlled trial to test the impact of a modified VHW care model on medication adherence, CVD risk, and cost.

**Trial registration:**

ClinicalTrials.gov, NCT06819839, retrospectively registered 27 October 2024.

## Introduction

Cardiovascular diseases (CVD) cause significant morbidity and mortality among conflict and crisis-affected populations worldwide [1, 2]. Evidence-based guidance for the management of CVD among populations affected by prolonged conflict and displacement is needed [3]. Community health worker (CHW) interventions have demonstrated effectiveness in screening for and treating non-communicable diseases in low- and middle-income countries and in stable displacement settings, but have not been tested in regions of active conflict [3–5]. Armed conflict settings present distinct implementation challenges that fundamentally impact care delivery, including severe mobility constraints, disrupted supply chains from attacks on health facilities, and workforce attrition and safety concerns that limit communication and supervision [6–9]. These conflict-specific challenges are distinct from stable low-resource settings and require evidence-based adaptive strategies. This is especially true for chronic, non-communicable diseases, that historically have received lower priority in emergencies, and require distinct infrastructure such as referral networks with advanced care capacity, and longitudinal health information systems that are particularly challenging to implement in conflict settings [10]. 

CVD is the leading cause of death in Myanmar [11, 12]. The most recent national study found that 3 in 4 adults 25–64 years old had one or more CVD risk factors, including hypertension (HTN) (26%), dyslipidemia (57%), and current smoking (26%) [13]. Myanmar has struggled to achieve high coverage of evidence-based interventions to diagnose and treat CVD risk factors [14]. The proportion of hypertensive individuals with controlled systolic blood pressure (< 140mmHg) is 12% or less; and uptake of statins and aspirin is less than 10% among individuals with a history of CVD or a calculated ten-year CVD risk over 30% [13, 15, 16]. Household surveys in 2019 among rural conflict-affected populations in eastern Myanmar found high prevalence of HTN (35%) and current smoking (39%), and less than 14% of hypertensive individuals had taken blood pressure medications in the previous two weeks [17]. 

The 2021 military coup reignited conflict throughout the country and worsened longstanding challenges with Myanmar’s health system, including chronic underinvestment, strained resources, and limited access [18]. The coup also precipitated a mass walkout among government health staff and increased attacks on health workers, further degrading health services nationwide [19]. These limitations are notable in areas of chronic conflict such as Karen State, where regional local health organizations (LHOs) have provided cross-border health aid to populations affected by conflict for over 50 years, including a basic package of primary care. These programs are largely delivered by teams of medics, CHWs, and village health workers (VHWs). Medics undergo 2 years of training and apprenticeship and provide clinical care, including diagnosis and treatment of basic health conditions [20]. CHWs undergo 6 months of training to support medics and conduct promotion and prevention activities through village outreach. VHWs are village residents historically trained and equipped to perform discrete tasks related to a health project, such as malaria testing and treatment. LHOs have developed and scaled care models for medics, CHWs and VHWs to deliver malaria and reproductive health services at the village level; in contrast, treatment of CVD risk factors is facility-based [21, 22]. Given the likelihood of continued prolonged conflict, populations in Karen State may need to rely on LHO-provided services for years or decades to come—making it imperative that stable, evidence-based strategies be produced for this setting.

We describe the findings of a feasibility study to explore a VHW-led care model intervention to identify those with risk factors for CVD and link them to longitudinal healthcare in conflict affected regions of Karen State, Myanmar. This study was carried out by an international non-governmental organization (iNGO), whose name is not included out of security concerns for participating staff, in collaboration with a regional LHO, the Karen Department of Health and Welfare (KDHW) that provides healthcare to individuals in Karen State.

## Methods

This feasibility study was carried out over two months in three villages, in partnership with three local clinics. It represented Phase 2 of a three-part study designed to develop and test a VHW intervention to screen and treat individuals with elevated CVD risk. Phase 1 utilized multiple qualitative interviews, facility surveys and group workshops to develop a causal loop diagram, which was then used by the iNGO and local partners to co-design the VHW care model [23]. Following the feasibility study, Phase 3 is hybrid type 1 implementation study consisting of a cluster randomized control trial (cRCT) to test the impact of the care model on medication adherence, equity and cost, and explore implementation domains across 13 villages in Karen State. This study was approved by the George Washington University Institutional Review Board (IRB) and a local Community Ethics Advisory Board (CEAB).

### Study intervention

The VHW care model included the following components: community sensitization and mobilization, population census and screening, a confirmatory visit for study enrollment and monthly follow-up VHW home visits – a detailed description of the intervention is available in Table 1. Individuals eligible for enrollment had HTN, diabetes, calculated CVD risk > 10% per WHO CVD risk charts, or history of heart attack or stroke [24]. 

**Table 1:** VHW CVD Feasibility Study Intervention

Intervention phase	Description
Protocol Development Approach	The clinical protocol used in this study is the result of an independent process developed by the local LHO partner to increase alignment with WHO HEARTS 2020 guidance, with a medium-term goal to roll out the new protocol throughout all partner clinics. The clinical protocol was developed, reviewed and approved with the active participation of clinician-trainers affiliated with the implementing LHO as well as with Mae Tao Clinic, a regional training hospital, and were vetted by independent LHO clinical staff (including physicians) to ensure that medics felt comfortable with it prior to the start of the study
CVD Guidelines	The protocol was grounded in the World Health Organization HEARTS 2020 technical package of cardiovascular disease management in primary healthcare, with modifications to optimize alignment with local guidelines and healthcare infrastructure. CVD history was defined as patient-reported history of stroke or heart disease, or positive responses to the Rose Angina questionnaire or a stroke symptom screening. Specific diagnostic criteria were outlined for HTN, diabetes mellitus, and calculation of 10-year CVD risk using WHO non-laboratory-based charts. Treatment of HTN included stepwise initiation of three antihypertensive medications (amlodipine, losartan, and hydrochlorothiazide). Metformin was the initial treatment for type 2 diabetes. Statins were recommended for those with diabetes, history of CVD (stroke or heart attack), and those with 10-year CVD risk > 10% risk per the WHO calculator. Aspirin was recommended for those with a history of CVD. Beta-blockers were recommended for those with a history of heart attack. All enrolled patients received a package of non-pharmaceutical interventions including education and counseling on modifiable behavioral risks such as smoking cessation, medication adherence, and lifestyle changes. Criteria for urgent referral for those with severe or acute cardiovascular symptoms were outlined
Staff Selection and Training	VHW selection criteria were as follows: adult over 18, able to read and write Burmese, and able to travel to and from their village to the regional clinic once per month. Each VHW underwent a 7-day training consisting of studying basic concepts of CVD physiology, practicing measurement of blood pressure, blood sugar, height and weight, developing health communication skills for behavioral counselling, and learning to collect and record data from their visits in the VHW logbook. All VHWs were required to pass a competency evaluation including basic knowledge of CVD risk factors, complications of CVD, and an ability to carry out study procedures. Similarly, medics affiliated with each of the three selected clinics were asked to undergo a 5-day training that included a refresher on the LHO clinical protocol for treatment of hypertension, diabetes, and CVD risk factors; study procedures and referral pathways; and VHW supervision. Medic performance was assessed using case scenarios and deemed acceptable by trainers but not formally scored. This training was designed and delivered by a team of physicians from the iNGO and the partner LHO, who also oversaw the delivery of the intervention
Village Selection	The Phase 2 Feasibility Study was designed to facilitate inclusion of selected villages in the experimental group of the Phase 3 cRCT, given the potential for armed conflict to disrupt future study activities. In preparation for the cRCT and prior to the feasibility study, all villages in the catchment area of each of 6 clinics were matched 1:1 or 2:1 into pairs and triplets according to approximate size, travel time from the respective clinic, and presence or absence of an existing VHW. Three village triplets – one from each clinic area – were purposively selected based on the presence of a suitable VHW, accessibility during the rainy season, and relative accessibility during conflict. Within each triplet one village was selected at random to receive the intervention during the feasibility study; unselected villages (2 in each triplet) could later either be randomized to an experimental and a control group, or could both serve as matched control feasibility study village
Community Sensitization and Enumeration	Following a community sensitization and education session to introduce this feasibility study to village leaders and the village health committee and to enlist their support, village leaders and the VHW conducted house-to-house visits to enumerate all individuals 40 years or older in the village. This enumeration procedure followed routine practice of our partner LHO to conduct a census of all individuals in their catchment areas. The intention was for renumeration results to provide an updated list of adults at least 40 years old so that VHWs could mobilize them several days prior to screening events
Community Screening	All individuals over 40 in each village were invited to a central location for a CVD screening event. This screening was conducted by one medic, CHW, and VHW affiliated with the nearest clinic. After informed, written consent, participants were invited to provide basic demographic information, medical history (diagnosis of or treatment for CVD, HTN, diabetes, or tobacco smoking), and screening for self-reported pregnancy. Pregnant women were excluded from the study. Each participant had their height, weight, and blood pressure measured. Those with a history of heart attack, stroke, diabetes, HTN, or those with a body-mass index over 23 had a random blood glucose checked. All individuals found to have the following were asked to undergo a confirmatory visit in 1–2 weeks with a medic: self-reported history of heart attack, stroke, HTN or diabetes, elevated blood pressure > 140/90, blood glucose > 200, calculated WHO CVD risk score over 10%
Confirmatory Visits	All individuals eligible for a confirmatory visit were visited by a medic and VHW 1–2 weeks later either at home or at a designated community location. The purpose of the confirmatory visit was to confirm diagnoses of diabetes and HTN for those with elevated random blood glucose (> 200) and elevated blood pressure (> 140/90) during the initial community screening. During the confirmatory visit, each individual provided separate written informed consent. Demographic information and medical history were confirmed, and repeat blood pressure, weight and height were recorded. A pregnancy test was performed for all women under 50;pregnant women were excluded. Individuals with a history of diabetes or random blood glucose over 200 during the community screening had a repeat random blood glucose measured. Individuals were also asked the Rose Angina questionnaire and stroke screening questionnaire.(37) Individuals found to have confirmed HTN (blood pressure > 140/90 on both screening and confirmatory visit), diabetes (random blood glucose > 200 on screening and confirmatory visit), calculated CVD risk over 10%, or history of heart attack or stroke were invited and consented to participate in the study. Those who agreed were enrolled in the feasibility study and scheduled to receive a VHW follow-up visit in 3–6 weeks. Study enrollees were also asked additional questions related to household assets, medication adherence using Morisky’s scale, health service utilization, and symptoms of depression. Medics then provided each individual with appropriate medications according to the CVD study protocol for management of diabetes, hypertension, and CVD
VHW Follow up Visits	All study enrollees were assigned to a VHW from their village for follow up visits approximately every 4 weeks, tracked using a paper logbook. During these visits, VHWs measured blood pressure, blood glucose for those with diabetes, evaluated medication adherence using a single question screen and pill count, and recorded any medication side effects or acute conditions (including symptoms of heart attack, stroke, heart failure). VHWs also provided smoking cessation or medication adherence counseling as appropriate
Joint review of VHW logbooks by medics and VHWs	Within 2 weeks of each follow-up visit, VHWs traveled to local clinics and met with medics to share data from their visits and discuss each patient. Medics, after reviewing information provided, could decide to continue medications and provide a refill, adjust dosages, initiate new medications, or opt to visit the patient in their home for in-person evaluation. All decisions were documented in the VHW logbook
Study Conclusion	The feasibility study was considered concluded after each study enrollee had received two visits from their VHW. Participants continued to receive monthly VHW visits with ongoing data collection until enrollment in the cluster randomized controlled trial
CVD Protocol Updates (post-feasibility study)	Following the protocol deviations described in this manuscript, the VHW care model protocol was simplified: the updated VHW care model to be tested in the cRCT would focus exclusively on treatment of hypertension, diabetes, and increased CVD risk (with statins). Individuals with a history of CVD were referred for treatment by experienced clinicians; the VHW care model continued to provide pressure medications and statins, though deferred treatment with beta-blockers and aspirin to referral providers

### Data collection

All data from screening and confirmatory visits was securely collected on password-protected tablets using Kobo Collect, an open-source data collection and management platform [25]. Three questionnaires were developed to facilitate data entry and study-relevant calculations for the screening, confirmatory visit, and VHW visits. During the screening, the Kobo tool generated study IDs for each patient, performed calculations of body-mass index and CVD risk score based on entered data, and displayed alerts for severely abnormal blood pressure or blood glucose readings, with recommendations to repeat measurements or seek immediate on-site medic evaluation prior to continuing. During the confirmatory visit, the Kobo tool retrieved patient medical history from screening for medics to review and clarify, identified those with new HTN or diabetes diagnoses based on recorded measurements, and assisted with determination of study eligibility. It was also pre-loaded with names and dosages of the CVD study medications being used, with recommendations for new prescriptions displayed based on the patient’s diagnosis and the CVD treatment protocol outlined in Table 1. VHW visit data were collected in a paper logbook using a structured format for each visit component. Every month, data from the VHW logbook were entered into Kobo Collect by KDHW staff using a structured questionnaire and transmitted encrypted for analysis.

All three Kobo questionnaires were circulated to study staff and supervisors for review and feedback, then subsequently translated to Burmese and Karen, with back-translation to ensure accuracy. All VHWs and medics underwent a two-day training around using the tools, entering findings and uploading data. Given limited internet availability in the study villages, screening and confirmatory visit data were entered and stored locally, then uploaded weekly to an encrypted server. Data were cleaned and checked for duplicates, missing values, and CVD risk calculation errors on a weekly basis.

### Patient safety

At the time of screening and confirmatory visits, medics, VHWs and CHWs were trained to ask about symptoms that could indicate medical emergencies, such as new chest pain or new neurological symptoms. Blood pressure measurements above or below predetermined thresholds (systolic blood pressure < 90 or > 180, diastolic blood pressure < 50 or > 110, blood glucose < 60 or > 300) prompted urgent evaluation by a medic at the time of recording to determine if further care was needed. At the confirmatory visits, medics recorded and reviewed all medication prescriptions, as well as any new prescriptions being made. A selected chart review was planned to review medications and new prescriptions for a random subset of patients at the end of the feasibility study, as well as any patients lost to follow up. During each follow-up visit VHWs elicited participant symptoms of acute cardiovascular conditions or medication side effects. The VHW did not make diagnoses or medication changes; rather, they gathered information, counseled on adherence and delivered medications. Symptoms consistent with medication side effects or CVD complications warranted immediate communication with a medic, when available, or referral to the clinic or hospital. Referrals were recorded on paper forms by VHWs and communicated to senior clinicians who followed up with referred participants and reviewed medical records, when available. All emergent or urgent referral information was also reported immediately in a deidentified fashion to study PIs and respective IRBs.

A root cause analysis (RCA) was conducted in response to protocol deviations. The RCA was designed to understand why deviations occurred and to identify opportunities to minimize the likelihood of future deviations. The RCA included in-depth review of existing qualitative safety data, confidential discussions and in-person group meetings with the research team and implementing partner leadership, clinicians and support staff, and detailed review of all medication prescriptions using a structured form.

### Feasibility evaluation

This study was designed in accordance with established guidance on conducting feasibility and pilot studies for implementation trials and employed a mixed-methods approach [26–28]. The primary aims were evaluating viability of study protocols, assessing recruitment potential, and exploring acceptability, fidelity, and participant adherence to the intervention. Specific feasibility domains explored, along with targets, are summarized in Table 2.

**Table 2:** Feasibility Domains, targets and achievements

Variable	Feasibility target	Achieved / comment
Time to screen each individual and per village	20 min/individual	Achieved
Time to conduct community screening in each village	3–5 days per village	Achieved
Did patients understand screening consent and processes?	patients understand and are comfortable with screening consent and processes No perceptions of harm identified.	Achieved
What proportion of total over 40 screened?	Greater than 90%	Unknown due to low quality census data; subsequent census protocol revised
What proportion of those screened underwent confirmatory visit? What proportion could not be reached? What proportion refused when asked to undergo confirmatory visit? What proportion declined to provide consent for the study once eligibility was confirmed?	aim for no more than 5% loss at each stage	Achieved – 100% eligible screening participants attended confirmatory visit ; 100% eligible for longitudinal follow-up consented to VHW home visits
How long does the confirmatory visit/consent process take per patient and per village? How feasible/useful/reasonable are confirmatory visit processes?	confirmatory visit and consent should take less than 45 min/patient	Achieved
Did patients understand confirmatory visit and enrollment consent processes?	Patients understand and are comfortable with confirmatory visit and enrollment processes: points of confusion/concern will be addressed for main study	Achieved
What proportion of those enrolled in the study remain in the study at one month?	90%	Achieved – 96.8% at 2 months
Among those enrolled, what proportion initiated evidence-based therapies before the end of the feasibility period?	60%	Not achieved (57.4%) due largely to lack of experience with statin eligibility; target exceeded for BP medications (82.9%)
Of all evidence-based therapies for which subjects were eligible at baseline, what proportion were subjects adhering to after one month / at their most recent visit? (similar to primary outcome of the cRCT)]	30%	Achieved (36.6% at 2 months)
Are patients doing what they are asked to do? (e.g., take medication, be seen by a VHW, travel to clinic when required, etc.)	Study processes, including VHW visits, clinic referrals, medic visits, etc. are acceptable to patients. Medications are acceptable to patients.	Achieved
Are VHWs able to deliver the intervention as designed? Why or why not? (treatment specific fidelity rate: key components are the visits, measuring blood pressure and glucose when needed, measuring adherence and appropriately referring/screening for side effects)	VHWs can over 90% of the time deliver the intervention as designed	Achieved for 3 stated key components (97% completed 2 visits, 100% had blood pressure and blood glucose measurements taken per protocol, 100% had adherence measured at both visits) Side effect screening was performed, but medication adverse events were not immediately identified, prompting root cause analysis. These protocol deviations were sufficiently important clinically that substantial modifications to clinical guidelines and study procedures were made
How long does VHW visit take?	each visit should take less than 1 h	Achieved
How do VHWs feel about/interact with the forms? Are these understood? Filled out correctly?	VHWs generally can fill forms out and find them manageable, correctly filled in greater than 95% of the time.	VHWs correctly filled in information; however, the original logbook was physically too large and the format was redesigned
Quality of care: Are medics able to reliably follow the clinical protocol as designed? (treatment specific fidelity rates)	Medics follow protocol greater than 90% of the time, and can provide solid clinical rationale for deviations	Not achieved. Protocol deviations led to substantial changes to clinical guidelines and study procedures; training was augmented with directly observed care of mock and real patients
How do medics feel about forms (logbook, medic/VHW assessment forms, referral forms, etc.). What is useful/not useful about these? Too burdensome, not enough detail? How long does each form take to fill out?	medics can fill forms out in reasonable manner, have a good understanding of what forms are for, and do not find these inordinately burdensome	Achieved; feedback led to modifications to the VHW logbook as above
Are medics able to engage in VHW CVD program? Why or why not, and what are barriers/facilitators?	Medics engage in VHW program, trust information relayed to them, and feel the program improves patient care	Achieved, However, medics cautioned about possible opportunity cost for other clinic tasks
How do patients feel about interactions with VHWS/Medics/Clinic?	Patients feel safe and supported by the program, and able to access services needed	Achieved
What is the perception of feasibility by the organization (KDHW, iNGO, others? )	Broad perception of feasibility of program, after review of information provided above	Achieved, following root cause analysis of protocol deviations
Preliminary estimate of effect size of the care model on planned primary outcome of the cRCT (adherence to blood pressure and statin medications among treatment eligible participants), to inform sample size calculations for the cRCT	Feasibility study results should not be inconsistent with original power calculations for the cRCT.	Partially achieved. Change in primary outcome (high adherence to statins and BP medications) was felt to be biased by initial misunderstanding of statin eligibility that could not be corrected due to protocol deviations. Magnitude of change in adherence to blood pressure medications exceeded expectations (80%)
Health provider training	VHWs and medics attend all training sessions, with good self-reported understanding of CVD content, protocols, and	Not achieved. Clinicians expressed desire for additional training and support, which was developed and delivered prior to initiation of the cRCT

Quantitative metrics included number of patients at each stage (screening, confirmatory visit, and VHW visits 1 and 2), patient demographics (age, sex, household wealth, prevalence of HTN, diabetes and CVD in the sample population, depression and food security screenings, and number and type of new prescriptions) [29–31]. The planned primary outcome for the cRCT was medication adherence, defined as being prescribed at least one medication in each clinically appropriate medication class, as well as self-reported “moderate” or “high” adherence to prescribed medications (using the 8-item Morisky adherence scale) [32]. Adherence in this feasibility study was defined using the 8-item Morisky scale at study enrollment and as a single-item assessments at each VHW visit, defined as an answer of ‘never’ or ‘rarely’ to the question “How often do you have difficulty remembering to take all your medication(s)?”. The medication adherence denominator was all patients eligible for treatment. Other outcomes of the feasibility study included blood pressure and blood glucose at each intervention stage, calculated 10-year risk of CVD, and number of patients receiving CVD-related counselling. Safety-related variables included loss to follow-up, study withdrawal, urgent/emergent referrals, medication errors or protocol deviations, and prevalence of medication-related adverse effects.

Qualitative evaluation occurred through structured interviews and focus group discussions. Focus groups were conducted for each study village at two timepoints – after the screening event and after confirmatory visits. Each focus groups consisted of the medic, CHW and VHW assigned to the village, and elicited feedback around the feasibility of study tasks, perceptions of reach and acceptability, and areas of improvement to streamline workflows. All medics (*N* = 3) and VHWs (*N* = 3), as well as implementing LHO partner leaders (*N* = 2) also participated in individual interviews at the conclusion of the feasibility study. Structured interviews were also conducted with 9 study participants, one per village at each of three time points: after screening, after confirmatory visits, and at feasibility study conclusion. Participant interviews assessed understanding of intervention aims and consent processes, solicited feedback and evaluated the time and resources required for study participation. Patients who declined to participate in screening, to enroll, or withdrew were also asked to participate in a brief interview to identify reasons. All interviews were carried out by the iNGO or KDHW in the individual’s preferred language (Karen or Burmese). Interviews were audio recorded and subsequently deidentified, transcribed and translated prior to analysis. Transcripts were evaluated using a directed content analysis approach [33]. Initial coding categories were identified through review of relevant implementation theory and existing literature, and additional coding categories were added iteratively. After all transcripts were separately coded by two study personnel, they met to discuss any areas of discrepancy and summarize findings across each of the domains assessed, along with 1–2 representative quotes.

## Results

### Participant retention and screening characteristics

**Fig. 1:**
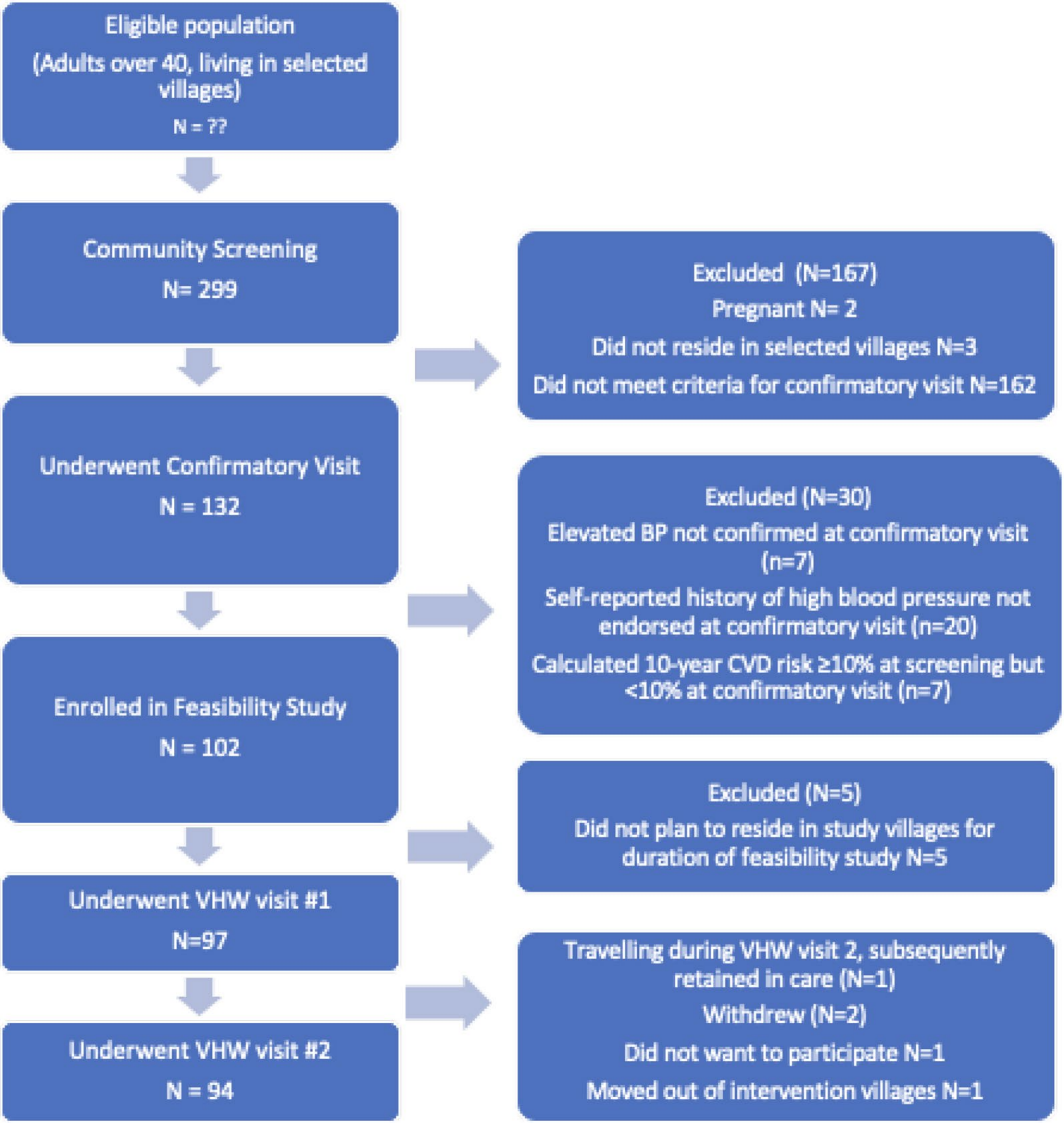
Flowchart of patients through intervention stages

Figure 1 shows participants in each intervention stage. Following selection and training of VHWs and medics and community sensitization, 299 individuals presented for screening across the three villages. Village census procedures performed by village leadership produced inaccurate results; subsequent enumeration of village residents for Phase 3 included direct participation by study team members. After exclusions, 294 were screened; 103 (35.0%) were male, mean age was 56.4 (range 40–95), and the majority (*N* = 172, 58.5%) reported current tobacco smoking. Six met criteria for diabetes (2.0%), 47 met initial criteria for HTN (16.0%), and 70 (23.8%) had 10-year CVD risk > 10%.

All 132 (44.9%) individuals eligible for a confirmatory visit were successfully reached, and all 102 eligible for the VHW care model consented to study enrollment. Subsequent review found that 5 individuals were ineligible for due to residence outside the village, thus 97 participants (33.0% of those who underwent screening) enrolled in the VHW care model. All 97 were visited by the VHW in their homes after month one (visit #1), and 94 (96.9%) participated in visit #2. Follow-up continued after the 2-month feasibility study period, with 92 (94.8%) of participants retained in care for visit #7.

### Characteristics of VHW care model participants

Table 3 summarizes characteristics of care model participants (*N* = 97), 35 of whom were male (36.1%), with a mean age of 66.5 (SD 13); 62(63.1%) were smokers. Most enrollees (*N* = 59/97, 60.8%) were from the poorest national wealth quintile, and 55/97(56.7%) were illiterate. Only 14/97(14.4%) reported receiving care for CVD in the past year, and travel time for CVD care was generally less than 2 h(78.6%). The most common reason for study eligibility was CVD risk > 10%(72.2%), followed by HTN(48.5%) and diabetes(6.2%). Among 47 individuals who met original study criteria for HTN, only 35 met WHO criteria of two elevated blood pressure measurements or current treatment;12 reported being told by a clinician they had HTN but were not on treatment and did not have elevated blood pressure during the screening and confirmatory visits. These participants were monitored monthly for 28 weeks; none had two or more elevated blood pressure readings.

**Table 3:** Characteristics of Individuals Enrolled in Monthly Village Health Worker Visits

Total patients enrolled	*N*	%
	97	100.0%
Male	35	36.1%
Age		
40–49	16	16.5%
50–59	7	7.2%
60–69	23	23.7%
70–79	37	38.1%
> 80	14	14.4%
Smoker	62	63.9%
Household Income		
Less than Ks 25,000	69	71.1%
Ks 25,000 – Ks 100,000	16	16.5%
Ks 100,000 – Ks 200,000	9	9.3%
Over Ks 200,000	3	3.1%
Myanmar Wealth Quintiles (EquityTool)		
Poorest	59	60.8%
Poor	24	24.7%
Medium	7	7.2%
Wealthy	7	7.2%
Wealthiest	0	0.0%
Education level		
Illiterate	55	56.7%
Primary education (Under 5th standard)	22	22.7%
Secondary education (under 9th standard)	10	10.3%
Higher education (till pass matriculation exam)	6	6.2%
Post graduate level (University)	1	1.0%
Monastery Education only (No specific standard)	3	3.1%
Positive depression screening (PHQ-2)	2	2.1%
Food Insecurity Experience Scale (FIES)		
Food secure (0–3)	88	90.7%
Moderate food insecurity (4–6)	9	9.3%
Severe food insecurity (7–8)	0	0.0%
Received prior CVD care (last 12 months)	14	14.4%
Travel time to receive CVD care (*N* = 14)		
< 30 min	5	35.7%
30 min to 2 h	6	42.9%
2 h to 6 h	2	14.3%
> 6 h	1	7.1%
Reported history of CVD	16	16.5%
Reported history of Stroke	13	13.4%
Reported history of MI	3	3.1%
Reason for study eligibility		
HTN	47	48.5%
DM	6	6.2%
CVD Risk > 10%	70	72.2%

### Medications & baseline adherence

Twenty-one patients (21.6%) reported taking at least one medication for CVD at the time of study enrollment, though only 14 reported receiving CVD care in the past year (14.4%). Of the 35 patients eligible for HTN medication, 18 reported taking an antihypertensive; 3 of 6 patients with diabetes reported taking metformin and only one of 75 eligible patients reported taking a statin. At the time of study enrollment, self-reported adherence to medications among those with a prior statin or antihypertensive prescription (*N* = 19) was low (36.8%) or moderate (52.6%); 2 reported high adherence (10.5%). Sixty-eight new prescriptions were made by medics for 52 participants during confirmatory visits; the most common prescriptions were for amlodipine(*N* = 27), losartan(*N* = 10) and atorvastatin(*N* = 15). Only 20% of those eligible for a statin prescription (*N* = 75) received one.

### VHW visits and outcome evaluation

Table 4 demonstrates outcome measures the beginning and end of the feasibility study, along with data from extended follow-up through visit #7. The proportion of patients receiving evidence-based treatment (statins or antihypertensives) was 23.2% at study enrollment and 57.3% at the end of the feasibility period. Medication adherence among those eligible for statin or HTN therapy(*N* = 82) was 2.4% at baseline and 36.6% at the end of the study period, and for HTN alone(*N* = 47) increased from 37.1% to 60%. Among participants with new prescriptions(*N* = 52), 49 (94.2%) received medication adherence counselling and 47 (90.4%) received refills at visit 2.

**Table 4:** Feasibility Study Outcomes

Number enrolled	At study enrollment	End of feasibility study (VHW visit #2)	Study continuation (VHW visit #7)
	97	94 (96.9%)	92 (94.8%)
Received smoking cessation counselling (among smokers)	N/A	36/62 = 58.1%	38/62 = 61.3%
Received diet and lifestyle counseling (among all enrollees)	N/A	72/94 = 76.6%	81/92 = 88%
Receiving evidence-based treatment for CVD (statins or antihypertensives, among eligible participants*)	19/82 = 23.2%	47/82 (57.3%)	46/82 (56.1)%
Moderate or high adherence to CVD medications (statins or antihypertensives)**	2/82 = 2.4%	30/82 = 36.6%	21/82 = 25.6%
Mean and SD blood pressure (SBP/DBP, among those with HTN, *N* = 47*)	142.7 (17.8)/86.6 (13)	127.3 (15.4)/78.1(10.2)	121.6 (12.8)/ 76.3 (8.4)
Blood Glucose (among those with DM, *N* = 6)	247.2 (108.9)	307.7 (148)	194.8 (58.7)
10-year CVD Risk mean (SD) (among all enrollees)	15.6 (9.3)	13 (9.2)	12.2 (9.4)

CVD = cardiovascular disease; SBP = systolic blood pressure; DBP = diastolic blood pressure; HTN = hypertension; DM = type 2 diabetes; SD = standard deviation

* The Feasibility Study enrolled *N* = 12 individuals who reported a history of hypertension (being told by a clinician they had “hypertension, or elevated blood pressure”) who were not taking antihypertension medications and did not have two elevated blood pressure readings. During 7 months of follow-up none of these 12 individuals had more than a single elevated blood pressure reading while not taking antihypertensive medications and thus did not meet clinical criteria for hypertension

** Defined by answer of ‘never’ or ‘rarely’ to a single-item question “How often do you have difficulty remembering to take all your medication(s)?”, with a denominator of all patients eligible for treatment (*N* = 82)

**Fig. 2:**
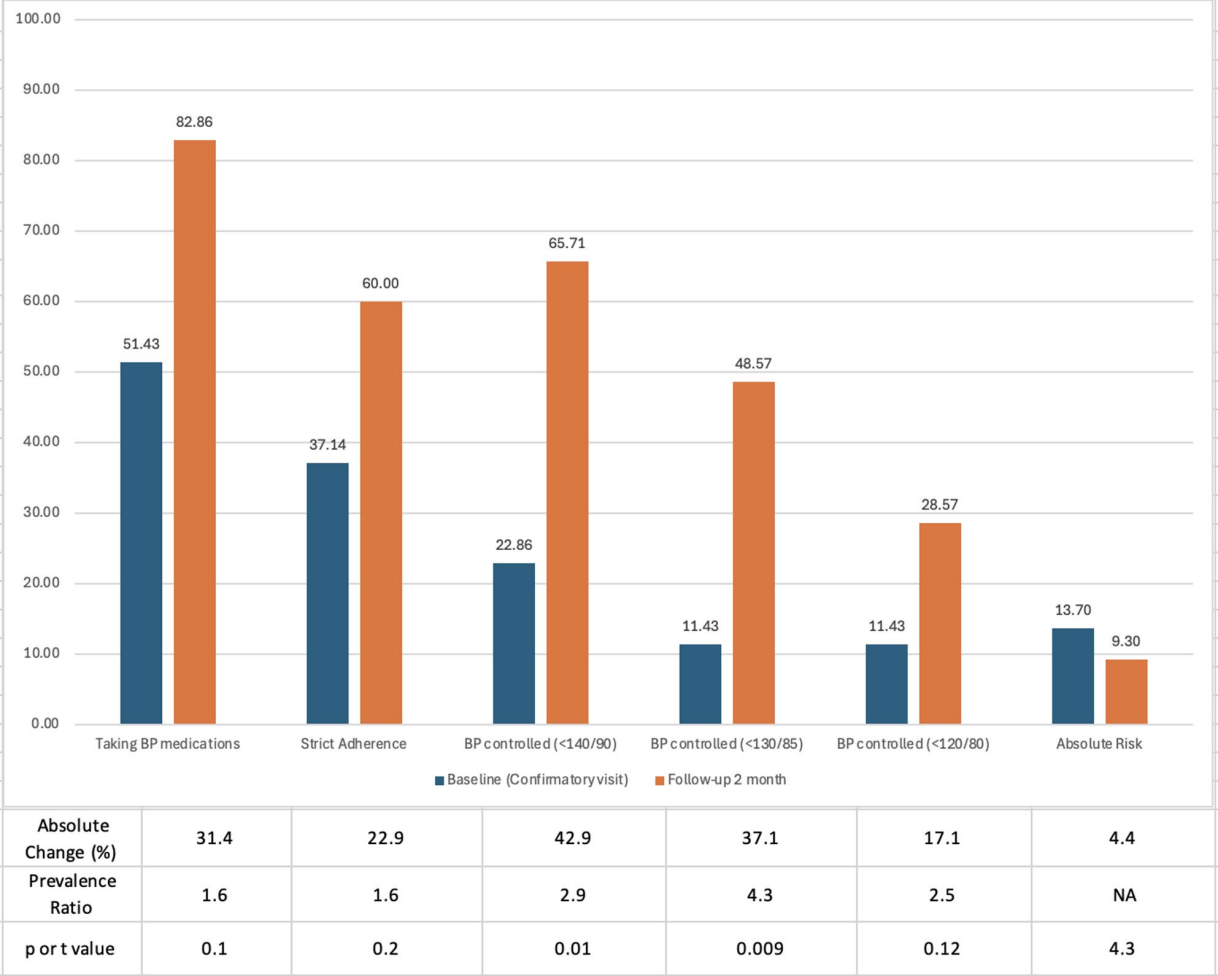
Hypertension Treatment Cascade. Among the subset of enrollees with HTN (*N* = 47), the proportion of patients achieving each phase of the hypertension treatment cascade, from time of study enrollment (blue) to the end of the feasibility study (orange), along with absolute change in percentage and prevalence ratios, where appropriate

Figure 2 demonstrates the HTN treatment cascade with absolute and relative changes in access to medications, adherence, and blood pressure control between the time of study enrollment and the conclusion of the feasibility study (VHW visit #2).

### Safety-related outcomes and protocol deviations

Three of 294 patients (1.0%) during the screening event and 2 of 132 patients during the confirmatory visit (1.5%) were referred for urgent evaluation by on-site medics for abnormal-range blood pressure or glucose, with no adverse events reported. Six enrolled participants (6.2%) were affected by protocol deviations resulting in prescription errors.

Five participants with a history of stroke were inappropriately prescribed atenolol when the electronic data collection tool recommended atenolol due to incorrect coding - the CVD protocol included atenolol for those with a history of heart attack, not stroke. The prescribing medics did not recognize the error, and recognition was delayed until the study team reviewed the VHW logbooks. Several participants experienced transient lightheadedness and others experienced mild hypotension (SBP 90–100mmHg). All participants were subsequently evaluated by a senior clinician on the study team; all incorrectly prescribed atenolol was discontinued. Clinical assessment did not reveal persistent harms.

One participant had their amlodipine prescription renewed despite reporting lightheadedness while taking amlodipine. The VHW correctly elicited and recorded the symptom in the VHW logbook; however, the medic refilled the amlodipine prescription without first evaluating the participant in person, as dictated by the CVD protocol. The participant’s symptoms were brought to the attention of the study staff during review of an interview transcript with a participant who described symptoms of lightheadedness associated with taking amlodipine. The interviewer was not a clinician and had not flagged the concerning symptoms; thus, this case was only addressed after one of the principal investigators read the qualitative transcript several days later. By the time the VHW rechecked the blood pressure, the participant had discontinued amlodipine and was symptom free.

As a result of these events, the study halted participant enrollment and medication initiation; any future dose titrations would be handled by a medical doctor. A root cause analysis was performed and all patients on study medications were personally evaluated by an on-site medical doctor using a structured form to review appropriateness, safety, and protocol alignment of all medications. Participants reporting any medication side effects were told to discontinue medications until evaluation by a medical doctor or medic. All enrolled patients received notification of the protocol deviations and mitigation strategies, and all provided written consent to remain in the study and continue monthly VHW visits. A report summarizing these events was compiled and filed with the appropriate IRB and CEAB.

### Qualitative interviews, focus group discussions & root cause analysis

Due to overlapping themes, findings from the RCA are presented together with qualitative analysis of transcripts from interviews and focus group discussions. Interview and focus group participants and stakeholders expressed broad support for the VHW-based model and its potential to deliver high quality CVD care; they also highlighted challenges faced by study personnel at different stages of the intervention. Representative quotes for each stage, along with lessons and modifications of the care model to be tested in the cRCT, are summarized in Table 5.

**Table 5:** Illustrative quotes from Qualitative Analysis and Lessons Learned for the Care Model to be tested in a Cluster Randomized Controlled Trial

Feasibility study phase/domain	Source	Quote	Lessons learned/ updates made to the VHW care model
Population screening	VHW, focus group discussion	“As for us, the fixed site is better. But every participant did not come here and door to door data collection may be better. But we told some people that we would be here for screening today and please come. But they went to the farm for harvesting and they can’t come. I say, if we make the screening day for 3 to 4 days, not just 2 days, they will come, I think. Door to door data collection is ok but some are busy and some sleep at the farm.”	Plan to avoid harvest season when scheduling population-based screening Alternative dates were not possible given funding period so additional days were scheduled to reach villagers harvesting crops
	Study participant, interview	“I’m so glad for your coming. No nurse here and it is hard for medications.”	
Confirmatory visit	Medic, focus group discussion	“The main issue is the naming. We searched with the names to know their [clinic ID] for the enrollment. Their real names, the names in the documents and in the Raw Data are not the same because of the spelling. Searching ID is fast but searching a name to get an ID is quite hard.”	Lookup function for Kobo Toolbox was revised with extensive input and testing from end users
	Study participant, interview	“(VHW) will visit once in a month or in 20 days, to measure blood pressure, to ask some questions and provide my medications monthly. That is what they said…The points I like is that we, the rural people, got difficulties to go to clinic. I’m glad that you came here and provide healthcare.”	
VHW visits	VHW, interview	“I’m also one of the villagers and sometimes the patients don’t listen to me. Patients believe more in what doctor said. They listen. Not all the patients. So, I want you to talk to some patients. If I talk alone, it doesn’t work.”	Reinforced with VHW that medics can and should conduct home visits for non-adherence and related reasons
	Study participant, interview	“It is great. VHW works hard. I don’t know about other people but a lot of benefits to me. If I went to clinic for medications, I spent some money and the whole day for medications. Now, it is pretty good for patients.”	
Clinical Protocol	LHO senior staff, interview	“The capacity of these medics for non-communicable diseases is somewhat limited, since they were more focused on communicable and symptomatic diseases. Since the area of cardiovascular disease is quite wide, including its wide drug range and difficulty in diagnosis, it is challenging to handle it even for….[anyone]….in a low-resourced setting, often needing us to draw conclusions based on the patient’s history instead of conducting further investigations. Therefore, the medics at the field level may have some weaknesses in managing CVD. When we interviewed them in the earlier stage, they admitted that they have limited exposure to managing non-communicable diseases and prescribing cardiovascular drugs. They may require more training and close supervision.”	Medics, CHWs and VHWs trained on additional CVD modules on disease-related symptoms, medication side effects Clinician knowledge evaluated and care quality assessed using direct observation and checklists during care for simulated and real patients
	Medic, interview	“This protocol is new to me and not quite familiar because we got a few patients. So, a few mistakes occurred when I used it”	
Protocol Deviations	LHO senior staff, interview	“It is quite challenging to fix errors that are identified only after checking the data. There were similar cases with the prescription of atenolol drugs in [redacted] where we only noticed the mistake after a month….We should not hastily proceed with screening activities solely relying on clinic medics. Having a responsible individual to manage and supervise the screening and confirmation processes in each village is crucial.”	Experienced clinicians (senior medic and physicians) were present for population-based screenings to handle large flux of villagers

#### Screening

Study participants were generally accepting of and grateful for the opportunity to receive screening for CVD risk factors and reported understanding of consent processes. VHWs and medics reported comfort with performing basic screening procedures, measuring blood pressure and glucose, and asking screening questions, though a few noted difficulties with tablet-related workflows, including uncertainty in how to return to data entry after receiving a warning message and taking appropriate safety steps. Study personnel expressed a range of opinions about the ideal logistics of village screening. Most felt that a fixed gathering area was the best approach, but recommended either longer availability for screening (3–4 days), avoiding the busy harvest season, or coordinating among other village meetings to improve turnout. Many patients had to be visited individually, which was time-consuming, either because they were homebound or busy working in the fields. Subsequent screenings provided prior notice and sequentially mobilized groups of households for screening.

**Confirmatory visits and study enrollment**: Study participants reported satisfaction with the confirmatory visit and comfort with study enrollment and consent, but many had to wait about 2 h to be seen. Medics and VHWs felt comfortable with carrying out visit procedures, measurements, and consent processes, though matching names to study IDs was considered difficult and time-consuming, primarily because many patients went by multiple names, spellings were variable in both Karen and Burmese, and not all participants knew if they had a pre-existing ID. Some of the questions for study enrollees around income and food security were considered too personal to ask in public. The low rate of statin prescriptions among eligible participants was attributed to medic/VHW/CHW inexperience with CVD-risk based indications for statins, and a misunderstanding that guidelines require a comorbid CVD risk factor be present for individuals 70 years and older. The misunderstanding was identified upon the first VHW logbook review, but supplemental training could not be completed prior to the protocol deviations and subsequent moratorium on new prescriptions.

Findings informed several modifications to confirmatory visit procedures: the clinic registration lookup was streamlined and incorporated multiple names, small groups of participants were mobilized sequentially to reduce wait times, the treatment suggestions offered by the tablet software were updated and extensively tested, and physicians or senior medics participated in all clinical decisions related to referral and treatment initiation.

**VHW visits**: Study participants were consistently grateful for the services provided by VHWs, particularly delivery of medications and facilitation of care closer to home. VHWs reported feeling comfortable with conducting home visits, recording relevant information, and advising patients to seek care in the clinics when appropriate. Their workload was considered reasonable, though some mentioned having to make multiple trips to reach study participants. Some reported that counselling on medication adherence seemed more effective when done by more senior clinicians. Logbooks were noted to be too large, and the format was revised prior to the cRCT. Medics felt that entering data from the VHW logbooks into the electronic tool was time-consuming; the task of data entry subsequently shifted to CHWs.

**Joint review of VHW logbooks by medics and VHWs**: Clinic staff identified delays hindering uploading, cleaning and eventual review of data that delayed timely identification of actionable symptoms or medication changes. Furthermore, the timeline to communicate changes to patients could be delayed due to unstable communications infrastructure and insecure transportation routes. In response, mechanisms for afferent and efferent communication were streamlined and data entry software was modified to flag all symptoms potentially related to study medications (such as dizziness) for potential follow-up. Enhanced training and experience of VHWs and medics was recommended to increase fidelity to the protocol and decrease the need for actionable feedback from supervisors.

**Clinical protocol and trainings**: While the CVD protocol was developed in conjunction with local stakeholders, and all medics received training on diagnosis and management of CVD risk factors, multiple medics and LHO-based leaders noted limited experience providing longitudinal care for patients with CVD, which meant medics had limited familiarity with CVD medications and side effects. As a result of this feedback and in light of the protocol deviations several changes were made: the VHW care model would focus on statins and blood pressure medications; it would no longer provide beta blockers or aspirin. All participants with a history of stroke or heart attack would be referred to care by experienced clinicians; participants could elect to receive statins and antihypertensive medications from the study. Additional training modules were developed for medics and VHWs, and fidelity to the protocol was assessed by direct observation of mock and real patients, using a quality-of-care checklist.

## Discussion

This study demonstrates the feasibility and acceptability of a VHW-led CVD care model aimed at improving screening and access to care for cardiovascular disease risk factors in a conflict-affected region of Myanmar.

Screening procedures were generally performed successfully, considered acceptable by study participants, and resulted in no perceived or measured harm. Identifying the reach of the intervention was complicated by inaccuracies in census-taking procedures which left uncertainty around the true number of eligible participants. This was potentially reflected in the gender imbalance seen among those screened and ultimately enrolled, with men representing only 35% of those screened and 36.1% of those enrolled. The short duration of the feasibility study period resulted in an overlap of screening activities with the harvest season, when many villagers were outside the village. Implementing partners agreed to extend the number of screening days and to prioritize calendar periods outside of the harvest season to more effectively access working-age men.

Confirmatory visits were successfully performed but often took two hours or more when waiting times were accounted for in addition to reconciliation of medications, consent, prescription of new medications and completion of detailed demographic questionnaires. Future rollout of the care model could consider deferring potentially sensitive questions related to income, food security or depression until the first VHW home visit, or dropping several question items entirely, depending on program objectives. A consistent concern raised was around the accuracy and speed of matching patient names to study IDs. Developing accurate systems for patient identification to facilitate longitudinal care and integrate digital health records from different sources has long been a challenge in low-resource and conflict-affected settings [24, 34, 35]. The study team subsequently modified the lookup function of the tablet to accommodate additional family names and nicknames and ID numbers were provided to participants on printed cards; this dramatically increased efficiency and accuracy of the identification process.

Among those screened, prevalence of smoking was higher than other studies in Myanmar (58.5% vs. 22.5%), while prevalence of diabetes (2.0% vs. 7.9%) and HTN (11.9% vs. 29.9%) were lower [8, 31, 32]. Smoking is a major modifiable CVD risk factor and given its high burden in this population, it may be valuable to assess the effectiveness and costs of integrating augmented smoking cessation approaches into the VHW care model, such as more intensive counselling, nicotine replacement, or medical assisted treatments [36, 37]. Lower prevalence of diabetes is likely due to limited sensitivity of two random glucose measurements, limited prior access to diabetes screening, and lower rates of obesity in this remote rural population [38]. The lower prevalence of HTN may be due, in part, to previously documented urban-rural differences in HTN and our clinical criteria that utilized blood pressure measurements from two time points, unlike cross-sectional surveys that rely on a single time point [39]. Seven participants with elevated blood pressure during screening did not have elevated blood pressure during the confirmatory visit and thus did not meet our clinical criteria. On the other hand, as part of this feasibility study 12 individuals who reported being told by a clinician they had HTN were enrolled in VHW follow-up for an indication of “hypertension” despite having non-elevated blood pressure off treatment, in order to learn whether to include self-reported history of HTN in the future care model to be tested in the cRCT. After multiple measurements over 7 months none of the 12 individuals had two elevated measurements, and history of HTN was dropped from eligibility criteria for a confirmatory visit. Results suggest that self-reported history of high blood pressure is not an accurate measure of HTN in this region; the low specificity suggests the question may also bias studies of the HTN care cascade and may over-estimate “awareness of HTN”.

Access to prior CVD care or medications was low (14–21%), particularly for statins (4.3%); prior studies conducted in more stable regions and periods in Myanmar found 34–48% of patients with HTN were receiving treatment [14, 16, 40]. This discrepancy is consistent with chronic under-investment in health services in remote rural areas of Myanmar and the low priority of chronic disease care in humanitarian crises. This further underscores the potential value of the VHW care model to bridge a crucial gap in access to care for this highly vulnerable population.

Medication adherence improved during the feasibility period but remained low (26–37%) when counting individuals not prescribed medication as ‘non-adherent’. This in part reflects the early misunderstanding of statin treatment indications that resulted in low numbers of prescriptions for statins – higher adherence was seen among patients with HTN (60%). In Phase 1 of this study, stakeholders noted that many villagers lacked experience taking daily medications; our findings suggest many individuals with chronic disease in this population can adhere to treatment if system barriers can be overcome. Nevertheless, additional work is necessary to identify and address barriers and facilitators to medication adherence to reduce CVD risk.

Data collection tools, including Kobo digital tablet applications and paper-based VHW logbooks, were implemented successfully, but opportunities for improvement were identified. Tablet-based tools involved multiple complex components to facilitate screening such as safety messages, calculation of CVD risk scores, and redirection for possible data entry errors. Several instances arose where study personnel did not know how to respond to a given alert or proceed to the next section of evaluation. Errors in tablet-based protocol recommendations for beta blocker therapy contributed to prescription errors, and would have benefitted from a longer validation period or more systematic testing of case-based scenarios, which has informed multiple protocol updates for the cRCT. Although the tablet tools were intended to provide recommendations for possible treatment, the limited experience with CVD care among providers may have increased reliance on tablet-based recommendations, making it more critical that they accurately reflect the clinical protocol. In preparation for the cRCT the team conducted a detailed review of all tablet workflows to ensure clarity and alignment with simplified protocols, performed multiple rounds of pilot testing, and expanded clinical trainings to include case-based practice with mock- and real patients .

Based on this feedback, the clinical protocol was streamlined to focus on the highest-yield components of CVD care in this specific setting – namely, access to evidence-based CVD medications, smoking cessation, and medication adherence. While local LHOs and staff have been providing high-quality, critical health services for this population, CVD represents a relatively small portion of existing care. Study participants and personnel expressed near unanimous support for substantial and sustained investment to extend enhanced CVD-related training and supervision of direct patient care among clinical providers who until now have lacked opportunities to provide longitudinal chronic disease care.

This study is the first to examine the feasibility of a VHW-led intervention for CVD in a conflict-affected region of Myanmar. Results demonstrated low access to CVD care at baseline for this population, with promising trends noted in access to medications, adherence, and HTN control among study enrollees. The study has several limitations. Inconsistencies in village census data precluded accurate assessment of screening coverage at the population-level. In part because population-level coverage was not considered an essential indicator to measure during the feasibility study, the census task was delegated to village leaders. In contrast, to improve the accuracy of enumeration for the cRCT the census will be conducted by dedicated study staff. The Morisky scale that was used for adherence evaluation has recently been retracted, complicating interpretation of outcomes defined in part by potentially unreliable or inaccurate thresholds for “moderate-to-high” adherence [41]. Following retraction, the Medication Adherence Report Scale-5 (MARS-5) scale was selected to measure adherence for the cRCT [42]. The two-month study duration precludes assessment of sustainability or regression to the mean. The lack of control group, small sample size, and potential biases such as the Hawthorne effect and measurement variability mean that outcome measures should be considered preliminary and descriptive rather than evidence of intervention effectiveness, which will be evaluated formally in the upcoming cRCT. Early protocol deviations resulted in a moratorium on medication changes that limited assessment of the care model’s potential for iterative quality improvement. Villages initially were selected based on accessibility during the rainy season, which limits generalizability to more remote villages. Delayed implementation until the harvest season likely reduced screening coverage, particularly among younger men.

In conclusion, this study evaluated feasibility of a VHW care model to address CVD risk in conflict-affected populations in Myanmar. The study team used lessons learned to selectively apply CVD treatment protocols, modify digital tools, and develop additional training and supervisory tools to strengthen longitudinal CVD care capacity among local providers. Results of Phase 2 Feasibility Study informed the design of the Phase 3 cRCT to test the impact of the VHW care model on CVD adherence, equity and cost.

## Data Availability

The datasets generated and/or analyzed during the current study are not publicly available out of concern for patient privacy and safety.

## Abbreviations

CVD: Cardiovascular disease
CHW: Community health worker
HTN: Hypertension
VHW: Village health worker
LHO: Local health organization
cRCT: Cluster randomized control study
CEAB: Community ethics advisory board
IRB: Institutional review board
RCA: Root cause analysis
